# Smith–Lemli–Opitz syndrome presenting as acute adrenal crisis in a child: a case report

**DOI:** 10.1186/s13256-018-1738-4

**Published:** 2018-08-10

**Authors:** Chamara Jayamanne, Sajith Sandamal, Kasun Jayasundara, Mayoorathy Saranavabavananthan, Sachith Mettananda

**Affiliations:** 1 University Paediatric Unit, Colombo North Teaching Hospital, Ragama, Sri Lanka; 2 Department of Paediatrics, University of Kelaniya, Ragama, Sri Lanka

**Keywords:** Smith–Lemli–Opitz syndrome, Adrenal crisis, Cholesterol biosynthesis

## Abstract

**Background:**

Smith–Lemli–Opitz syndrome is a rare autosomal recessive disorder of cholesterol biosynthesis which is characterized by multiple congenital malformations and global developmental delay. Here we report the case of a 3-year-old, previously undiagnosed, child with Smith–Lemli–Opitz syndrome presenting with acute adrenal crisis, which is an extremely rare and atypical presentation of this disease.

**Case presentation:**

A 3-year-old Sri Lankan Sinhalese boy without evidence of infection presented with circulatory collapse. He had a normal perinatal period; however, his early infancy was complicated by poor feeding, episodes of loose stools, failure to thrive, and several episodes of unexplained drowsiness. His weight, height, and occipitofrontal circumference were well below the third percentile. He had soft dysmorphic features that included microcephaly, bitemporal narrowing, upward slanting eyes, epicanthal folds, partial ptosis, broad nasal bridge, low set posteriorly rotated ears, high arched palate, and short neck. Marked hyperpigmentation was noted in perioral, buccal, and palmar areas. His pulses were rapid and low in volume and his systolic blood pressure was low. Initial resuscitation was performed by administering multiple crystalloid fluid boluses. A septic screen was negative. His blood glucose and serum bicarbonate levels were low and serum electrolytes revealed hyponatremia with hyperkalemia. Serum spot cortisol level was low normal and 17-hydroxyprogesterone level was low. Diagnosis of Smith–Lemli–Opitz syndrome and associated adrenal crisis was made based on clinical and biochemical features. Intravenously administered hydrocortisone was commenced to which he showed a marked clinical response.

**Conclusions:**

This case describes a rare and atypical presentation of Smith–Lemli–Opitz syndrome and highlights the importance of making early and accurate syndromic diagnoses in children with dysmorphism to avoid sudden and life-threatening complications.

## Background

Smith–Lemli–Opitz syndrome (SLOS) is a disorder of cholesterol biosynthesis which is characterized by multiple congenital malformations and global developmental delay. This rare autosomal recessive condition was first described by Smith *et al.* and has an incidence of 1:20000 to 1:70000 [1]. Due to defective steroid hormonogenesis, children with SLOS manifest variable degrees of deficiencies of adrenocortical hormones; however, acute adrenal failure is rare. Here, we report the case of a 3-year-old, previously undiagnosed, child with SLOS presenting with acute adrenal crisis. This case report describes a rare and atypical presentation SLOS and highlights the importance of making early and accurate diagnoses in children with malformations to prevent life-threatening complications.

## Case presentation

A 39-month-old Sri Lankan Sinhalese boy from a poor socio-economic background presented to the pediatric ward with circulatory collapse. He did not have fever, history of infection, or other identifiable focus of sepsis. He was born to non-consanguineous parents at 38 weeks of gestation with a birth weight of 2.5 kg and had an uncomplicated perinatal period. In early infancy he had problems of poor feeding, episodes of loose stools, and failure to thrive despite nutritional supplementation and had chronic constipation during the past 2 years. On further inquiry his mother described several episodes of unexplained drowsiness at times of minor infections which settled without interventions except for an episode 6 months previously which was associated with hypoglycemia that required a dextrose infusion.

**Fig. 1:**
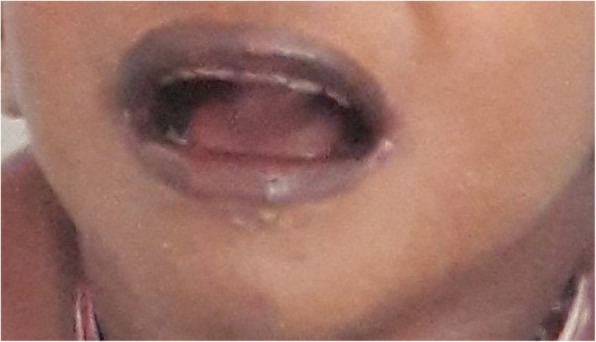
A photograph of the patient’s face demonstrating dysmorphic features and marked hyperpigmentation in the perioral region

Anthropometric measurements revealed: weight 6.9 kg (well below third percentile), height 76 cm (below third percentile), and occipitofrontal circumference 42 cm (below third percentile). He had dysmorphic features which included microcephaly, bitemporal narrowing, upward slanting eyes, epicanthal folds, partial ptosis, broad nasal bridge, low set posteriorly rotated ears, high arched palate, and short neck. Anterior fontanelle was still open. Marked hyperpigmentation was noted in his perioral, buccal, and palmar areas (Fig. 1). His fingers and toes did not show any abnormalities and his genitalia were normal. He had tachycardia, low volume pulse, and his systolic blood pressure was recorded as 50 mmHg. There were no abdominal masses or genital abnormalities. Hypotonia with reduced power (4/5) was noted in all muscle groups. Tendon reflexes and examination of eyes were normal. A development assessment by examining his developmental milestones revealed global developmental delay with a developmental age between 15 and 18 months.

His blood glucose (30 mg/dL) and serum bicarbonate (20 mmol/L) levels were low and serum electrolytes revealed hyponatremia (sodium 120 mmol/L) with hyperkalemia (potassium 7.1 mmol/L). Serum osmolality was 275 mosm/L. Renal and liver function tests and serum calcium, magnesium, and phosphate levels were normal. Blood and urine cultures were sterile and C-reactive protein level was within normal range. Serum spot cortisol level on admission was 133 nmol/L (normal 120–626) and 17-hydroxy progesterone level was 1.171 ng/dL (reference 3–90) suggesting failure of initial steps of steroid hormone synthesis. Serum cholesterol was normal (153 mg/dL). His condition did not permit us to perform an ACTH stimulation test before commencement of treatment. Ultrasound scans of his abdomen and brain, echocardiography, and electroencephalography were normal. His karyotype was 46XY. Genetic studies and 7-dehydrocholesterol (7DHC) levels were not performed due to unavailability.

Acute adrenal crisis was diagnosed based on characteristic biochemical abnormalities and SLOS was considered as the possible etiology based on dysmorphic features. The child was resuscitated with two 0.9% sodium chloride 20 ml/kg fluid boluses and hypoglycemia was corrected using 10% dextrose 3 ml/kg bolus. This was followed by intravenous 5% dextrose in 0.9% sodium chloride fluid infusion. Intravenously administered hydrocortisone 50 mg single dose was followed by 12.5 mg dose every 6 hours for the next 24 hours and he was started on intravenously administered cefotaxime. He showed a marked response to hydrocortisone with normalization of hyponatremia and hyperkalemia which did not require other specific treatment. After 24 hours, orally administered hydrocortisone 10 mg/m^2^ per 24 hours in three divided doses and orally administered fludrocortisone 0.1 mg daily were commenced. His parents were asked to give foods high in cholesterol (for example, eggs). Follow-up was arranged in the pediatric clinic with monthly measurements of weight, height, blood pressure, and serum electrolyte levels.

## Discussion and conclusions

SLOS is due to a deficiency of the enzyme 7DHC reductase, which catalyzes the final step in the cholesterol biosynthetic pathway [2]. Mutation in the gene that encodes this enzyme results in accumulation of 7DHC and low plasma cholesterol level. Over 150 mutations of this gene have been identified; the commonest of which is c.964-1G>C (IVS8-1G>C) mutation, which is a splice acceptor mutation [3]. In homozygous state, this mutation exhibits the complete phenotype of SLOS with major congenital malformations which include agenesis of corpus callosum, abnormal gyration of the brain, bilateral renal agenesis, oligohydramnios, severe lung aplasia, and cardiac defects (atrioventricular septal defects and aortic hypoplasia). The clinical severity of SLOS due to other mutations is variable and correlates to the degree of deficiency of 7DHC reductase enzyme levels. Less severely affected children live with minor physical anomalies, mild developmental delay, and autistic traits.

Cholesterol is an essential precursor of adrenal hormone biosynthesis. Deficiency of cholesterol in SLOS leads to defective synthesis of all adrenal cortical hormones, which results in adrenal insufficiency that is rarely so severe as to cause acute adrenal crisis as in this child. Most of the less severely affected patients do not exhibit features of adrenal failure; however, the degree of cholesterol deficiency which results in adrenal failure in SLOS is unknown [4]. Therefore, timely diagnosis and watchful anticipation is necessary for early identification of serious complications of SLOS.

In the patient described in this case report, the diagnosis of SLOS was not made at birth or early infancy due to subtleness of dysmorphism. The diagnosis was only established at the time when the child presented with severe hypovolemia due to adrenal failure. A normal serum cholesterol level does not exclude the diagnosis of SLOS because current laboratory assays of total cholesterol measure certain precursors of cholesterol especially 7DHC which is high in SLOS. In conclusion, this case describes a rare and atypical presentation of SLOS and highlights the importance of making early and accurate syndromic diagnoses in children with dysmorphism to avoid sudden and life-threatening complications.

## Abbreviations

7DHC: 7-Dehydrocholesterol
SLOS: Smith–Lemli–Opitz syndrome

## References

[1] Nowaczyk MJ, Irons MB. Smith-Lemli-Opitz syndrome: phenotype, natural history, and epidemiology. Am J Med Genet C Semin Med Genet. 2012;160C(4):250–62. 10.1002/ajmg.c.31343 23059950

[2] Porter FD. Smith-Lemli-Opitz syndrome: pathogenesis, diagnosis and management. Eur J Hum Genet. 2008;16(5):535–41. 10.1038/ejhg.2008.10 18285838

[3] Waterham HR, Hennekam RC. Mutational spectrum of Smith-Lemli-Opitz syndrome. Am J Med Genet C Semin Med Genet. 2012;160C(4):263–84. 10.1002/ajmg.c.31346 23042628

[4] Bianconi SE, et al. Adrenal function in Smith-Lemli-Opitz syndrome. Am J Med Genet A. 2011;155A(11):2732–8. 10.1002/ajmg.a.34271 PMC3488380 21990131

